# Synthesis, DFT Analysis, and Dyeing Performance of 4,4’-Dihyroxy-3-Substituted Azobenzene Disperse Dyes with Comparative Evaluation of Aqueous and DMF-Based Application Systems

**DOI:** 10.1007/s10895-026-04754-z

**Published:** 2026-04-20

**Authors:** Mohamed A. El-Rahman, Mai S. Alsubaie, Mohamed A. El-Atawy, Hussam Y. Alharbi, Majed S. Aljohani, Saad Alrashdi, Alaa Z. Omar, Ezzat A. Hamed, Reham O. El-Zawawy

**Affiliations:** 1 Chemistry Department, Faculty of Science, Alexandria University, Alexandria, 21231 Egypt; 2 Department of Chemistry, College of Science in Yanbu, Taibah University, Yanbu Governorate, Saudi Arabia; 3 Department of Chemistry, College of Science, Jouf University, Sakaka, Aljouf 72341 Saudi Arabia; 4 Chemistry Department, Faculty of Science, Damanhour University, Damanhour, 22511 Egypt; 5 Research Department, Health Sciences Research Center, Princess Nourah bint Abdulrahman University, Riyadh, Kingdom of Saudi Arabia

**Keywords:** Disperse dye, Azo dye, Aminophenol, Color strength, DFT, Fastness properties

## Abstract

A series of 4,4′-dihydroxy-3-substituted azobenzene disperse dyes (**2a–f**) were synthesized, fully characterized (FT-IR, ^1^H/^13^C NMR, CHN elemental analysis) and evaluated by DFT (B3LYP/6-31G(d, p)) to link electronic structure with dyeing performance. TD-DFT reproduces experimental λ_max_ with deviations ≤ 12 nm, supporting the computational model. Compound **2e** (3-CHO) shows the highest electrophilicity (ω = 4.6441 eV) and dipole moment (µ = 4.9366 D), while dye **2d** (3-COOH) delivered the highest color strength (K/S = 21.64 in 5% DMF–95% H_2_O), corresponding to a ≈ 46.3% increase relative to the aqueous + dispersant system (K/S = 14.79). Exhaustion (%E) and fastness data (wash, perspiration, scorch, light) indicate generally commercial-grade fixation for the series, with light fastness uniformly high (7–8). We show that modest DMF addition (5% v/v) improves color uptake for some substituents but can slightly reduce certain fastness metrics; therefore DMF is used here as a mechanistic probe (improves solubility/partitioning under controlled lab conditions) rather than a recommended scale-up solvent. The combined experimental, DFT analysis identifies structure property rules to guide design of disperse dyes with improved color strength and predictable application behaviour.

**Supplementary Information:**

The online version contains supplementary material available at 10.1007/s10895-026-04754-z.

## Introduction

Disperse dyes represent a specialized class of synthetic colorants specifically engineered for dyeing hydrophobic synthetic fibers, particularly polyacetate, and polyamide textiles. These water-insoluble organic dyes are characterized by their relatively small molecular size, planar structure, and absence of ionic groups, enabling them to penetrate the compact polymer matrix of synthetic fibers through high-temperature (HT) dyeing processes [1]. The improvement of disperse dyes, pretreatment of fibre and dyes before dyeing process, the use of different methods of dyeing such as the use carriers, leveling agent [2], solvent, microwave [3], ultrasonic conditions [4, 5], can provide excellent color fastness, vibrant hues, and superior performance characteristics that meet the demanding requirements of modern textile applications [6].

Azo dyes constitute the largest and most versatile class of synthetic colorants, accounting for approximately 70% of all commercially produced dyes worldwide [7, 8]. These compounds are characterized by the presence of one or more azo linkages (-N = N-) that serve as the primary chromophoric system responsible for their intense coloration and broad spectrum of achievable hues. The synthetic accessibility of azo dyes through classical diazotization and coupling reactions, combined with their structural diversity and excellent color properties, has established them as the predominant choice for textile, leather, paper, and plastic coloration applications [9]. Azo disperse dyes, in particular, combine the favorable dyeing characteristics of the azo chromophore with the substrate compatibility required for synthetic fiber applications [10], offering exceptional color strength, photostability, and thermal resistance that are essential for high-performance textile applications [11].

The successful application of disperse dyes requires the use of dispersing agents or alternative solvent systems to maintain stable dye dispersions and facilitate uniform color improvement [12, 13]. Traditional aqueous dyeing systems employ non-ionic dispersing agents such as DYEWELL-002, which reduces surface tension and prevents dye aggregation while maintaining compatibility with synthetic substrates. However, the growing interest in alternative dyeing methodologies has led to the exploration of organic solvents such as dimethylformamide (DMF) as both solvent and dispersing medium [14]. DMF offers several advantages including high dye solubility, elimination of dispersing agent requirements, and enhanced dye-fiber interactions due to its high polarity and thermal stability [15]. The dual functionality of DMF as a solvent-dispersant system presents opportunities for simplified dyeing processes while potentially improving color yield and fastness properties, though comparative studies are essential to validate these theoretical advantages.

In parallel with improvements to aqueous dyeing, a family of non-aqueous dyeing technologies has emerged as a promising route to eliminate wastewater and reduce the environmental footprint of textile coloration. These approaches include supercritical CO_2_ dyeing, silicone-based non-aqueous media (e.g., decamethylcyclopentasiloxane, D5 or linear silicone fluids), and low-water or two-phase hydrophobic media that solubilize disperse dyes and promote direct fiber partitioning without large aqueous effluents [16–19]. Recent pilot and laboratory studies demonstrate that non-aqueous systems can achieve high color yield and reduced effluent while introducing new formulation and fiber-surface issues (e.g., oligomer migration, solvent-mediated swelling and altered dye aggregation) that require different process controls than conventional baths.

Polyester fabrics, primarily composed of polyethylene terephthalate (PET), represent the most widely used synthetic textile material globally due to their exceptional durability, dimensional stability, wrinkle resistance, and cost-effectiveness [9, 20]. The highly crystalline structure and hydrophobic nature of polyester fibers present unique challenges for dyeing processes, requiring elevated temperatures (typically 120–140 °C) and specialized dye formulations to achieve satisfactory color development. The absence of polar functional groups in the polyester backbone necessitates the use of disperse dyes that can penetrate the fiber structure through physical interactions rather than chemical bonding [21, 22]. The dyeing mechanism involves the diffusion of dye molecules into the amorphous regions of the polymer during high-temperature treatment (HT), followed by physical entrapment upon cooling, making the understanding of dye-polymer interactions crucial for optimizing dyeing performance.

Density functional theory (DFT) has emerged as a powerful computational tool for predicting and explaining the electronic properties, reactivity patterns [23, 24], and structure-activity relationships of organic dyes. The use of DFT study in dye chemistry enables researchers to calculate fundamental molecular descriptors such as frontier molecular orbital energies, chemical hardness, electrophilicity indices, and molecular electrostatic potential surfaces that directly correlate with dyeing performance and color properties. Time-dependent DFT (TD-DFT) calculations provide accurate predictions of electronic absorption spectra, facilitating the rational design of dyes with tailored optical properties [25, 26]. The integration of theoretical calculations with experimental studies has proven invaluable for understanding dye-fiber interactions, optimizing molecular structures for enhanced performance, and reducing the time and cost associated with traditional trial-and-error approaches in dye development.

Despite the diversity of available dyeing technologies, the literature lacks controlled studies that directly compare aqueous dispersant-based dyeing with dispersant-free using the same dye structures and standardized dyeing parameters. Without such comparisons, it remains difficult to isolate the true impact of dispersants effects on dye aggregation, diffusion, and fixation behaviour. To address this gap, the present study synthesizes a series of 4,4′-dihydroxy-3-substituted azobenzene disperse dyes derived from 4-aminophenol and combines molecular-level theory with tightly controlled dyeing experiments. Specifically, we (1) characterize the electronic and spectroscopic properties of the dye series (UV–Vis, IR, ^1^H/^13^C NMR, and DFT/TD-DFT calculations), (2) evaluate dyeing performance on polyester using rigorously standardized protocols (identical bath ratio, dye load, heating/cooling rates, hold time, and pH) across two application media, aqueous with dispersant, and 5% DMF–water, (3) quantify colour strength, exhaustion and fastness under these comparable conditions to isolate the effects of dispersant versus solvent, and (4) correlate DFT-derived descriptors with experimental optical and dye-fiber interaction outcomes.

## Experimental

### General Method for Synthesis of Dyes 2a-f

#### Step 1: Preparation of diazonium

In a 250 mL capacity conical flask, a mixture of *4*-aminophenol (0.008 mol), 7 mL conc. HC1 and 14 mL water were stirred to clear solution and kept in cooling 0° to −5^o^ C in an ice bath. Add 5 g sodium nitrite into *p*-aminophenol hydrochloride solution dropwise with constant stirring. The reaction mixture shows the positive test of nitrous acid on starch-iodide paper (blue color is obtained on the potassium-iodide starch paper).

#### Step 2: Reaction of diazonium salt with coupling reagent

Add above prepared diazonium salt solution slowly to well cooled mixture of 2-substituted phenol (Namely; phenol, *o*-florophenol, *2*-chlorophenol, 4-hydroxy benzoic acid (salicylic acid), 2-hydroxy benzaldehyde (salicylaldehyde) and *2*-methyl phenol (0.006 mol) dissolved in 15 mL H_2_O and 0.3 g sodium hydroxide in 15 mL of water. The progress of the reaction was monitored by TLC and then crude dyes were filtered, washed with hot water for several times. Each crude dye was recrystallized from appropriate solvents (ethanol/DMF or ethanol/water, depending on solubility). The purity was confirmed by TLC and sharp melting points.

## **2a** [27], **2d** [28] and **2e** [29]

### (***E***)−3- Fluoro-4,4’-Dihydroxyazobenzene 2b

Brown crystals, 85% yield; m.p. 201–203 °C. IR (KBr): ῡ 3498 (OH), 3066 (sp^2^ =C-H) and 1598 (N = N) cm^−1^. ^1^H NMR (500 MHz, *d6*-DMSO): δ 7.84 (d, 2H, 2Ar-H), 7.61 (d, 2H, 2Ar-H), 7.35 (m, 2H, 2Ar-H) and 7.10 (s, 1H, Ar-H) ppm. ^13^C NMR (126 MHz, *d6*-DMSO): δ 163.84 (d, *J* = 249.0 Hz, C-F), 153.22, 151.28, 149.17, 144.49, 124.93 (d, *J* = 9.1, 1.5 Hz), 123.43 (d, *J* = 4.5 Hz), 118.26 (t, *J* = 3.4 Hz), 117.25 and 108.44 (d, *J* = 19.1 Hz) ppm. C_12_H_9_FN_2_O_2_ requires: C, 62.07; H, 3.91; N, 12.06%. Found: C, 62.14; H, 3.85; N, 12.12%.

### (***E***)−3- Chloro-4,4’-Dihydroxyazobenzene 2c

Reddish brown crystals, 88% yield; m.p. 184 °C. IR (KBr): ῡ 3244 (OH), 3021 (sp^2^ =C-H) and 1603 (N = N) cm^−1^. ^1^H NMR (400 MHz, *d6*-DMSO): δ 10.90 (s, 1H, OH), 9.49 (s, 1H, OH) and 8.27–6.49 (m, 7 H, 7Ar-H) ppm. ^13^C NMR (101 MHz, *d6*-DMSO): δ 136.24, 134.67, 130.59, 128.55, 127.34, 124.25, 120.35, 116.90, 115.68 and 115.43 ppm. C_12_H_9_ClN_2_O_2_ requires: C, 57.96; H, 3.65; N, 11.27%. Found: C, 58.05; H, 3.57; N, 11.32%.

### (E)−3-Methyl-4,4’-Dihydroxyazobenzene 2f

Orange crystals, 90% yield; m.p. 165–167 °C. IR (KBr): ῡ 3233 (OH), 3051 (sp^2^ =C-H), 2943 (sp^3^ -C-H) and 1602 (N = N) cm^− 1^. ^1^H NMR (400 MHz, *d6*-DMSO): δ 10.17 (s, 1H, OH), 9.42 (s, 1H, OH), 8.35–6.23 (m, 7 H, 7Ar-H) and 2.04 (s, 3H, CH_3_) ppm. ^13^C NMR (101 MHz, *d6*-DMSO): δ 135.73, 133.25, 131.94, 130.95, 130.72, 124.64, 116.32, 115.46, 115.32, 107.84 and 31.24 (CH_3_) ppm. C_13_H_12_N_2_O_2_ requires: C, 68.41; H, 5.30; N, 12.27%. Found: C, 68.48; H, 5.28; N, 12.34%.

## Dyeing Procedure and Post-Treatment

### Dyeing Procedure

Polyester fabric samples (2.0 g) were dyed by high-temperature exhaust (HT) dyeing in a 250 mL dyeing vessel using a bath liquor ratio of 20:1 (L: R). Dyeing baths were prepared at a dye concentration of 2.0% owf (on-weight of fabric). For aqueous dyeing the dispersing agent DYEWELL-002 was added at 1.0 g·L^− 1^. For the mixed solvent system, 5% (v/v) DMF in H_2_O was used with no additional dispersant. The initial bath pH of all systems was adjusted to 4.50 ± 0.10 (acetic acid) prior to heating. The dyeing program was run as follows: load the sample at 30 °C, heat to 130 °C at 2.0 °C·min^− 1^, hold at 130 °C for 60 min, then cool to 50 °C at 2.0 °C·min^− 1^. Agitation was continuous (mechanical stirring) throughout heating, holding and cooling. After cooling, the dyed samples were rinsed with warm water (3 × 100 mL) to remove superficial dye.

### Post-Treatment

Reduction clearing was performed to remove unfixed surface dye and improve fastness. Dyed samples were treated in 200 mL of aqueous solution containing sodium hydroxide (NaOH) 5 g·L^− 1^ and sodium dithionite (Na_2_S_2_O_4_, also called sodium hydrosulfite) 2 g·L^− 1^ at 60 °C for 10 min under gentle stirring. Immediately after reduction, samples were rinsed thoroughly with warm water, and dried at room temperature.

### Quality Checks and Reproducibility Measures

Bath temperatures were monitored and logged with a thermocouple; pH was measured before heating, at the holding temperature and after cooling. Dye exhaustion (%E) was calculated from pre- and post-dyeing bath absorbance values.

## Results and Discussion

### Chemistry

The syntheses of azo dyes exhibit an extensive range of hues, making them indispensable in various industries such as textiles, paints, plastics, and printing [30]. Azo dyes owe their vividness to a well-known synthetic route that involves the diazotization of aromatic amines, followed by coupling reactions with appropriate coupling components, such as phenols.

At the heart of this synthetic process lies 4-aminophenol **1**, serves as the precursor for countless azo dye structures. The synthesis begins with the conversion of 4-aminophenol into its diazonium salt through reaction with sodium nitrite in the presence of hydrochloric acid at low temperatures. Diazotization introduces a highly reactive diazonium group (-N ≡ N^+^) onto the aromatic ring, setting the stage for subsequent color-forming reactions.

**Scheme 1:**
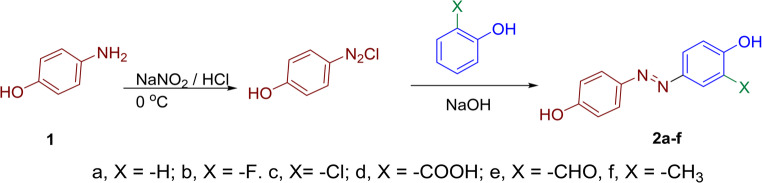
The synthetic pathway of disperse dyes **2a-f**

Once the diazonium salt of 4-aminophenol is obtained, it can be coupled with a diverse range of coupling components (2-substituted phenol, X = -H, -F, -Cl, -COOH, -CHO, -CH_3_) yields azo dyes **2a-f**, Scheme 1, which exhibit a dazzling array of colours depending on the nature of the 2-substituted phenol coupling component used. The careful selecting of different substituted phenols can achieve an extensive palette of shades. This versatility makes azo dyes highly desirable for applications where precise colour control is crucial.

The FT-IR spectra of azo dyes **2a-f** (a, X = -H; b, X = -F. c, X= -Cl; d, X = -COOH; e, X = -CHO, f, X = -CH_3_) revealed characteristic absorption of broad band at range ῡ 3498 − 3233 cm^− 1^ assigned to stretching vibration of hydroxyl group, while the aromatic C-H bonds appears as a weak peaks at 3066 − 3021 cm^− 1^. Furthermore, the appearance of medium intense absorption peak at ῡ 1603 − 1598 cm^− 1^ is attributed to the azo group (N = N). The ^1^H NMR spectra of dyes **2a-f** showed deshielded exchangeable broad singlet signals in the range δ 10.90–9.42 ppm corresponds to two -OH protons. Expansion of ^1^H NMR peaks in range δ 8.35–6.23 ppm are assigned to the aromatic protons. Additionally, dye **2f** (X = 3-CH_3_) showed weak peaks at 2943 cm^− 1^ for aliphatic C-H vibration in FT-IR and at 2.04 ppm in ^1^H NMR spectrum. These spectral results indicate that the structure of all products are 4, 4’-dihydroxy-3-substituted azobenzene **2a-f**, Table 1.

**Table 1:**
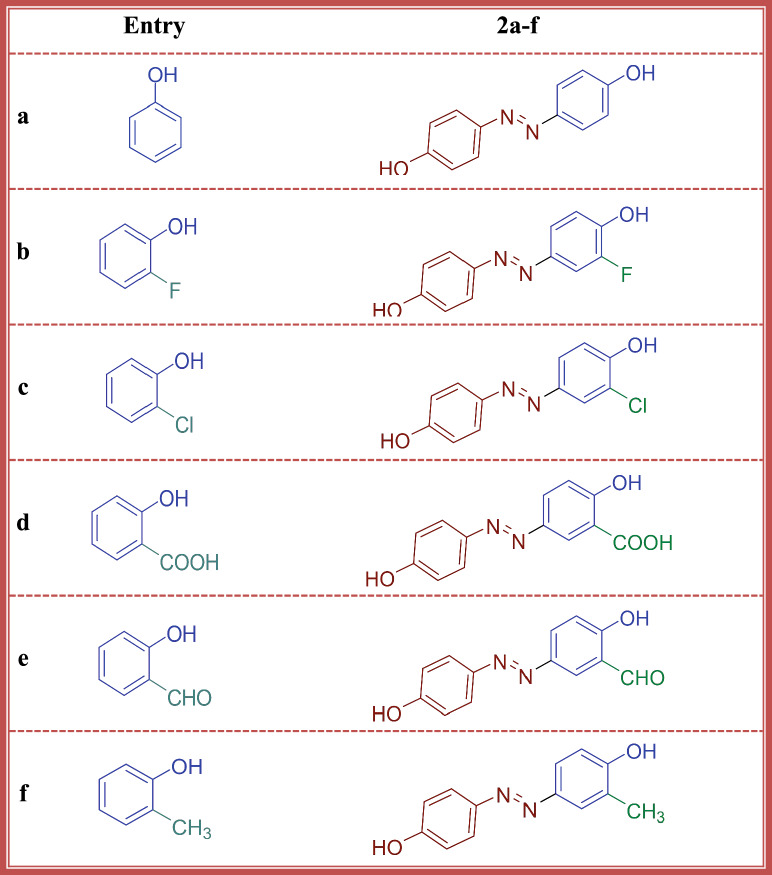
The coupler and the structure of dyes **2a-f**

### Electronic Sbsorption Spectra

**Fig. 1:**
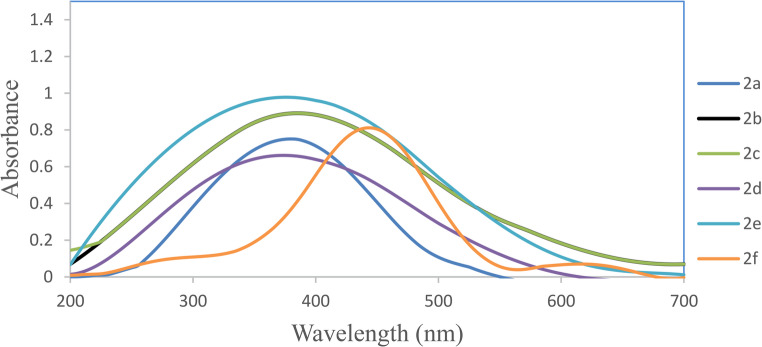
UV-Visible spectra of disperse dyes 2a-f in DMF

The electronic absorption spectra of the synthesized disperse azo dyes **2a-f** were determined using both experimental UV-visible spectroscopy in DMF and the investigated Time-Dependent Density Functional Theory (TD-DFT) calculations. The B3LYP functional with the 6-31G(d, p) basis set are used. The UV–Vis spectra (Table 2; Fig. 1) show λ_max_ values of 375–445 nm, with **2f** (445 nm) red-shifted by 65 nm relative to **2a** (380 nm). TD-DFT reproduces λ_max_ within ≤ 12 nm, confirming the computational model and supporting the substituent-dependent shifts (electron-donating 3-CH_3_ → bathochromic; electron-withdrawing groups → hypsochromic).

**Table 2:** Experimental and computed absorption maxima of dye **2a-f** in DMF, DMF/0.1 M NaOH and DMF/0.1 M HCl

Dye no.	Coupler	DMF λ (nm)		λ (nm)	
		Experimental	Computational	DMF + NaOH	DMF + HCl
2a	phenol	380	382	455	355
2b	2-florophenol	385	384	430	355
2c	2-chlorophenol	383	384	425	360
2d	2-carboxyphenol	375	379	455	330
2e	2-formyl phenol	375	370	440	330
2f	2-methyl phenol	445	457	470	380

The structural variations in the coupler components significantly influenced the absorption characteristics of the resulting dyes. The aryl moieties containing 4,4’-dihydroxy groups **2a** (X = H) exhibited an absorption maximum at 380 nm, serving as a reference point for evaluating substituent effects.

**Fig. 2:**
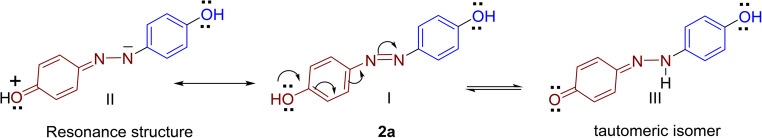
Possible resonance structures and tautomeric isomers of 2a

The possible resonance structures of compound **2a** (X = H) are structure II and its tautomer isomer III are shown in Fig. 2, both –OH substituent make the same structures. At first the introduction of substituent at position 3- deviates the 4-OH from the plane of the molecule and thus it’s effect becomes inductive only while the second -OH at position 4’ becomes responsible for the resonance and tautomerization.

**Fig. 3:**
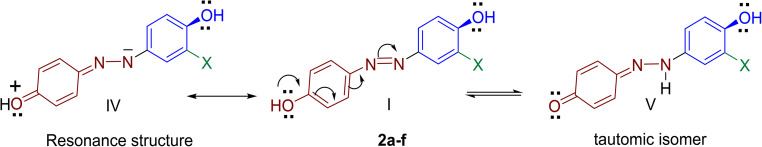
Possible resonance structures and tautomeric isomers of 2b-f

Therefore, the presence of halogen substituents in position 3-X resulted in a slight bathochromic shift like the 3-F (**2b)** and 3-Cl (**2c).** They displayed an absorption at 385 and 383 nm, respectively. This is presumably due to the stabilization of resonance structure II. The 3-COOH substituent **2d** and 3-CHO substituent **2 g** both exhibited hypsochromic shifts with absorption maxima at 375 nm, indicating that the presence of these groups in the ortho position with respect to the hydroxyl group may be (i) create intramolecular hydrogen bond interactions between both substituents that inhibit the resonance between the 4-OH substituent and the azo one and/or (ii) these groups are sterically hindered the 4-OH group causing them non-coplanar with the aromatic ring and prevent its delocalization of electrons reflecting no electronic transitions and in turn decrease in its absorption compare the parent dye **2a**. The most significant bathochromic shift was observed in the 3-CH_3_ dye **2f**, with an absorption maximum at 445 nm, representing a 65 nm red shift compared to the parent dye **2a** (X = H). This pronounced bathochromic effect can be attributed to the electron-donating nature of the methyl group, which inhibits the formation of resonance structure IV while it enhances the existence of initial molecule I and tautomer isomer V, thereby lowering the energy gap between ground and excited states, Fig. 3.

### Effect of Acid and Base Addition on Electronic Absorption Spectra of Dyes 2a-f

**Fig. 4:**
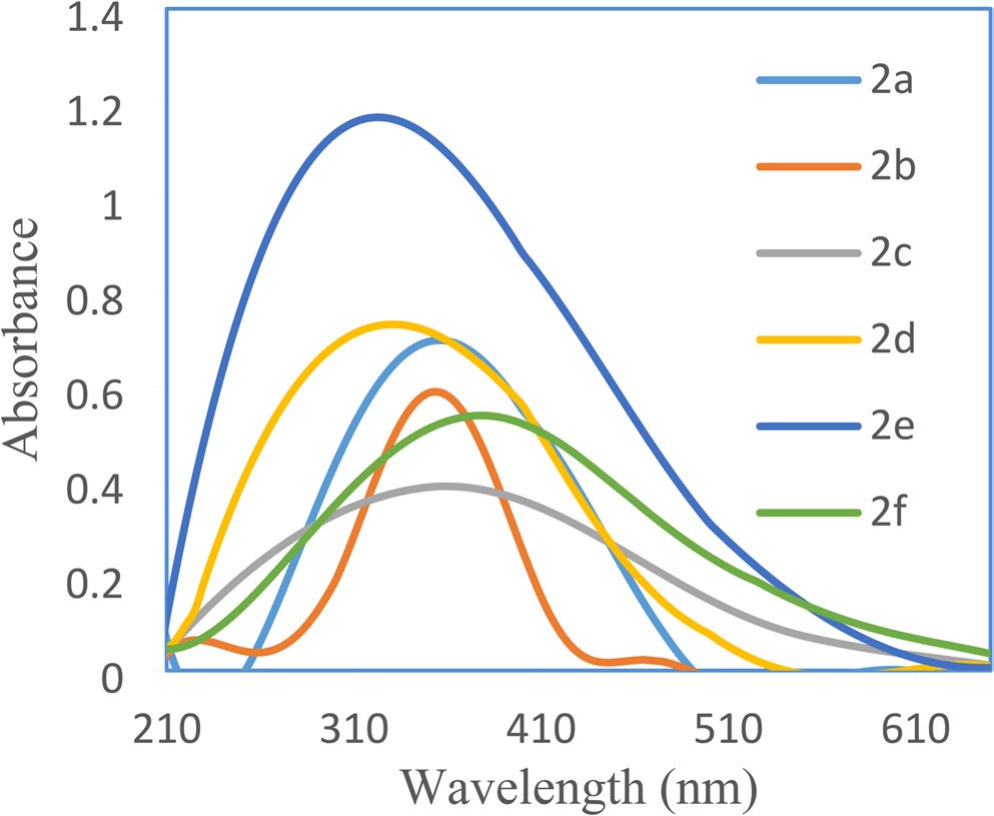
Absorption spectra of dyes 2a-f in DMF and 0.1 M HCl

**Fig. 5:**
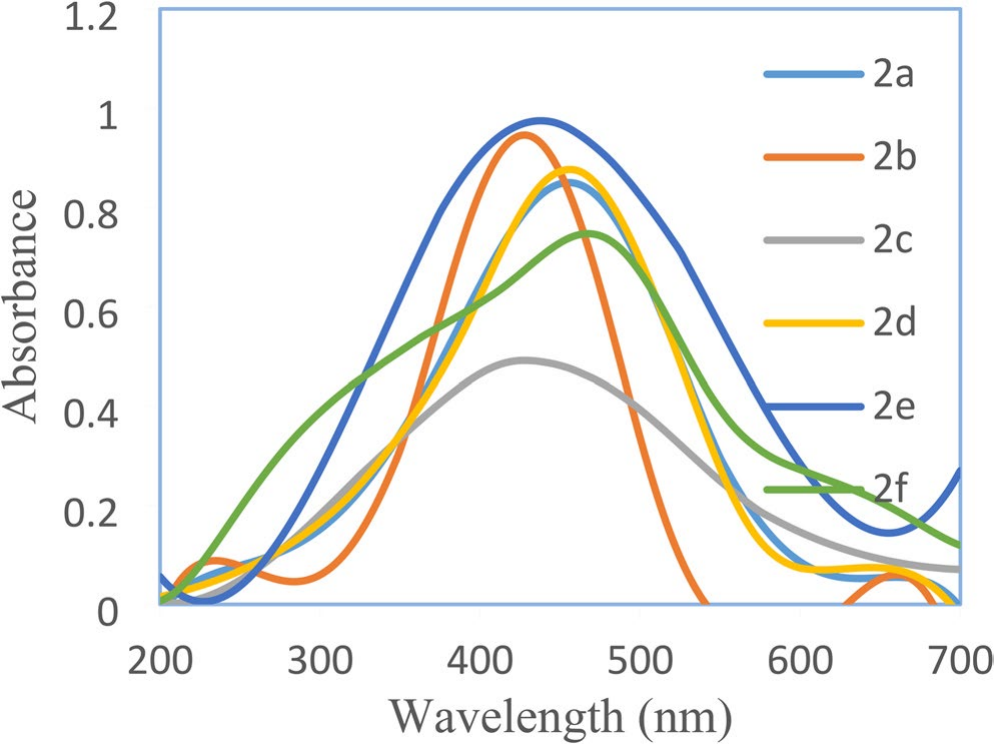
Absorption spectra of dyes 2a-f in DMF and 0.1 M NaOH

The pH-responsive behavior of the disperse azo dyes **2a-f** was systematically investigated by examining their electronic absorption spectra following the addition of 0.1 M NaOH and 0.1 M HCl to DMF solutions, Figs. 4 and 5.

Addition of 0.1 M NaOH produced bathochromic shifts of 50–80 nm across the series; notably **2a** and **2d** shift by 75–80 nm, acidification (0.1 M HCl) gives smaller blue shifts (25–45 nm), showing the response is strongest in basic media and supporting the dyes’ potential as colorimetric basic-range indicators (see Table 2; Figs. 4 and 5).

**Fig. 6:**
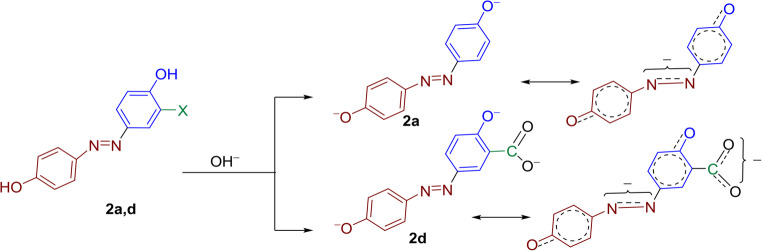
Deprotonation of disperse dyes **2a**and** 2d** under basic condition

Under basic conditions, The pronounced bathochromic response of **2a** and **2d** can be attributed to the formation of phenolate and carboxylate anions, respectively, which act as electron withdrawing than the neutral phenolic groups, thereby increase the stabilization of these anions and enhancing π-electron delocalization throughout the conjugated system from 4’-OH to the other aryl ring, Fig. 6.

The **2b** (X = 3-F) and **2c** (X = 3-Cl) exhibited moderate bathochromic shifts under basic conditions, with absorption maxima at 430 nm and 425 nm, respectively. The smaller magnitude of these shifts compared to the **2a** (X = H) suggests that the electron-withdrawing nature of halogen substituents partially counteracts the electron-donating effect of the phenolate anion, resulting in a more balanced electronic environment.

The dye **2e** (3-CHO) displayed an intermediate bathochromic shift to 440 nm under basic conditions, while **2f** (X = CH_3_) showed a more modest shift to 470 nm. The relatively smaller shift observed for **2f** may be attributed to the fact that this dye already has significant bathochromic absorption in its neutral form due to the electron-donating methyl group, leaving less ability for further red-shifting upon base addition.

**Fig. 7:**
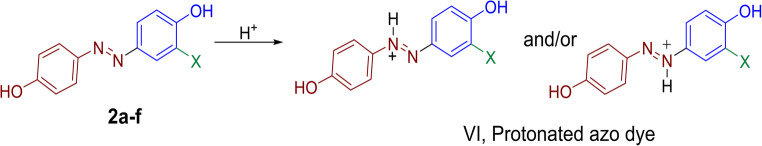
Protonation of disperse dyes 2a-f under acidic condition

In contrast, the addition of 0.1 M HCl resulted in hypsochromic shifts for all compounds due protonation of nitrogen atoms of the azo group, VI, Fig. 7, with absorption maxima λ_max_ shifting to shorter wavelengths. The magnitude of these blue shifts was generally smaller than the corresponding red shifts observed under basic conditions. The dye **2a** (X = H) and **2b** (X = 3-F) both showed shifts to 355 nm upon acid addition, representing hypsochromic shifts of 25 nm and 30 nm, respectively. The dye **2c** (X = 3-Cl) exhibited a similar pattern with a shift to 360 nm.

The most pronounced hypsochromic shifts under acidic conditions were observed for the compounds containing 3-COOH **2d** and 3-CHO **2e** both shifted to 330 nm, representing substantial blue shifts of 45 nm from their neutral absorption maxima. This enhanced acid sensitivity may be attributed to the presence of additional protonation sites or specific intramolecular interactions that inhibit delocalization of electrons under acidic conditions.

The dye **2f** (X = 3-CH_3_) showed a hypsochromic shift to 380 nm under acidic conditions, representing a decrease by 65 nm shift from its DMF only. The relatively large magnitude of this difference in shift correlates with the enhanced bathochromic absorption of dye **2f** in neutral conditions. This points out that the protonation of **2f** is proportionally related to prevent the delocalization of electrons and cutting out the extent of conjugation as in the neutral form.

The observed pH-dependent spectral behavior demonstrates the dual-responsive nature of the titled disperse azo dyes **2a–f**, with a significant pH-dependent spectral shift (red shift of 75–80 nm under alkaline conditions), making them highly promising colorimetric pH indicators, particularly in the basic range where the spectral changes are most pronounced and visually distinctive. The previous behavior of dyeing **2a-f** in acid-base media let us to study the dyeing process and examine dyed polyester fibre properties such as exhaustion values %E, colour strength K/S, colorimetric analysis and different types of fastness.

### Dyeing Performance and Dye-Fiber Interactions

The application of the synthesized disperse azo dyes **2a-f** to dye polyester fabrics was accomplished through the exhaust dyeing technique at high temperature (HT), which relies on the transfer of dye molecules from the dye bath to the hydrophobic polyester substrate under controlled conditions. Two distinct methodological approaches were employed to optimize the dyeing performance of compounds **2a-f**. The first approach utilized an aqueous dyeing system incorporating DYEWELL-002, a non-ionic dispersing agent specifically designed to maintain the stability of disperse dye dispersions. This dispersing agent functions by reducing the surface tension between the hydrophobic dye molecules and the aqueous medium, preventing the formation of large dye aggregates that would otherwise compromise uniform color development. The non-ionic nature of DYEWELL-002 ensures compatibility with the polyester substrate while providing sustained dispersion stability throughout the high-temperature dyeing process.

**Fig. 8:**
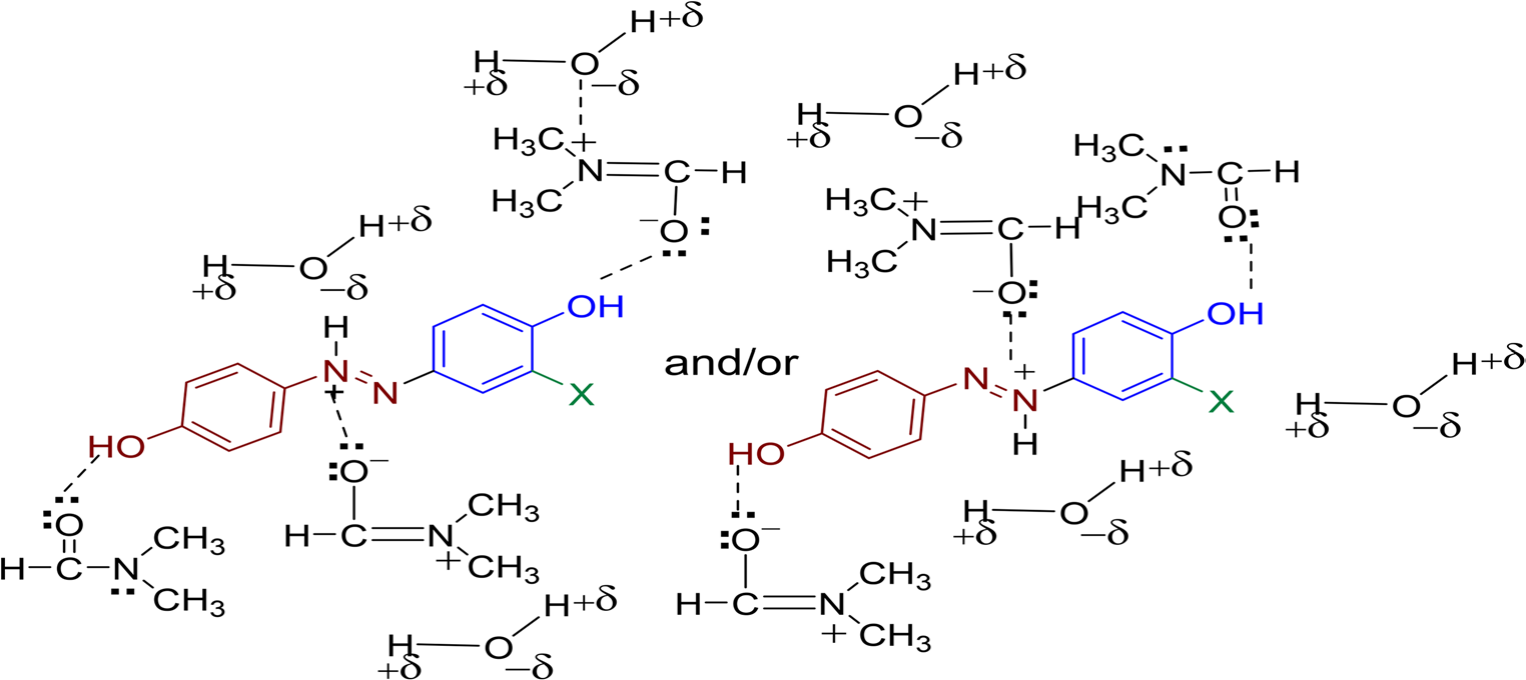
Possible interactions between dyes 2a-f with a mixture of DMF and H_2_O molecules

The second methodology employed dimethylformamide (95% H_2_O, 5% DMF) instead of pure water. DMF acts as both solvent and dispersing medium for the dyeing process. DMF demonstrates exceptional solvating capacity for disperse dyes due to its high polarity and ability to form favorable interactions with the protonated azo chromophore, Fig. 8. The dual functionality of DMF as a solvent-dispersant eliminates the need for additional dispersing agents while maintaining superior dye dissolution and distribution characteristics. The high boiling point and thermal stability of DMF make it particularly suitable for the elevated temperature conditions required for effective polyester dyeing.

Both dyeing methodologies were conducted under identical HT conditions following a standardized protocol optimized for disperse dye application.

The maintenance of optimal pH conditions represents a critical parameter in disperse dyeing, as the acidic environment facilitates dye dispersion and prevents unwanted side reactions [31]. The temperature-time combination provides sufficient driving force for dye diffusion into the compact polyester structure while avoiding thermal degradation of either the dye molecules or the polymer substrate. At the end of dyeing process controlled cooling to 50 °C was implemented to prevent thermal shock and maintain the integrity of the dye-fiber interactions established during the high-temperature phase.

Post-dyeing treatment procedures were essential for achieving optimal color quality and fastness properties. The dyed fabrics underwent thorough washing to remove surface-deposited dye particles and residual dispersing agents that could compromise color clarity and hand feel. The alkaline reduction process not only enhances brightness and clarity by removing surface impurities but also improves the overall fastness properties by ensuring that only properly fixed dye molecules remain within the fiber structure [32].

The mechanism of dye-fiber interaction for the synthesized azo dyes **2a-f** on polyester substrates encompasses three interconnected steps that collectively determine the final dyeing outcome.

The initial adsorption step involves the migration of dye molecules from the bulk dye bath to the fiber surface, driven primarily by hydrophobic affinity between the nonpolar segments of the dye molecules and the polyester surface, facilitated by the aromatic character, protonated structure VI of the azo dye and the polyethylene terephthalate polymer backbone [33]. The subsequent diffusion step represents the critical step where adsorbed dye molecules penetrate the amorphous regions of the polyester fiber structure, with the applied high-temperature conditions (HT) enhancing the mobility of polyester chain segments and creating transient voids that accommodate dye molecule penetration through thermally activated channels within the polymer matrix. The final fixation step establishes the intermolecular interactions that anchor the dye molecules within the polyester structure, ensuring colour permanence and fastness properties [34, 35].

The structural characteristics of dyes **2a-f** provide multiple interaction sites with the polyester polymer chains. The phenolic -OH groups and the protonated azo group can engage in hydrogen bonding interactions with the carbonyl oxygen atoms of the polyester ester linkages, while the extended conjugated system of the azo chromophore enables Van Der Waals interactions and potential π-π stacking arrangements with the aromatic terephthalate segments [36].

#### Properties of Fastness

The performance characteristics of polyester fabrics dyed with the synthesized azo dyes **2a-f** were systematically evaluated using two distinct application methodologies: aqueous dyeing with DYEWELL-002 dispersing agent and a mixture of H_2_O-DMF dyeing using the same condition. The fastness properties were assessed according to standardized testing protocols [37, 38], encompassing wash fastness, perspiration fastness under both acidic and alkaline conditions, scorch fastness on cotton and polyester substrates, and light fastness, with ratings on established scales where 5 represents excellent performance for most properties and 8 indicates superior light fastness [39], Table 3.

**Table 3:**
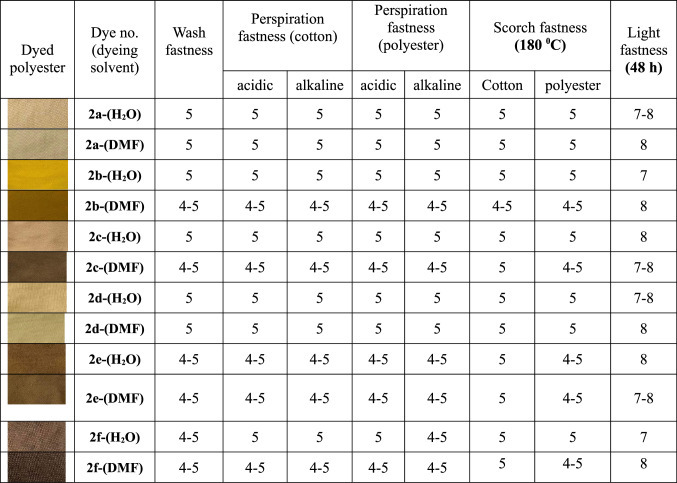
Fastness characteristics of disperse dyes **2a-f**usingaqueousdispersing agent DYEWELL-002 in pure H_2_O and 5%DMF-95% H_2_O

Comparative analysis of the wash fastness data reveals distinct performance patterns between the two dyeing methodologies and among the different dye structures. The parent dye **2a** demonstrated exceptional wash fastness ratings of 5 with both aqueous and a mixture of H_2_O-DMF dyeing methods, indicating robust dye-fiber interactions that resist detergent-induced removal regardless of the application technique. For example, the 3-F and 3-Cl derivatives **2b** and **2c**, respectively (achieved excellent wash fastness ratings of 5 with the aqueous method, though a slight reduction to 4–5 was observed when a mixture of H_2_O-DMF is using in the dyeing process, while compounds **2e** (X = 3-CHO) and **2f** (X = 3-CH_3_) consistently demonstrated good to excellent ratings (4–5) regardless of the dyeing method employed. The presence of 3-COOH group **2d** maintained excellent wash fastness (5) across both methods of dyeing, suggesting that the carboxylic acid functionality may contribute to enhanced dye fixation through additional hydrogen bonding interactions with the polyester substrate.

Perspiration fastness evaluations under both acidic and alkaline conditions revealed remarkably consistent performance across the entire dye series, with most compounds achieving excellent ratings regardless of the dyeing methodology employed. The dyes **2a** (X = H), **2c** (X = 3-Cl), and **2d** (X = 3-COOH) demonstrated outstanding perspiration fastness ratings of 5 under all test conditions when aqueous dyeing is used, indicating exceptional stability against hydrolytic degradation in sweat-simulating environments. The DMF-based dyeing method produced slightly reduced ratings (4–5) for compounds **2b** (X = 3-F), **2c** (X = 3-Cl), 2e (X = 3-CHO) and **2f** (X = 3-CH_3_), though these values still represent commercially acceptable performance levels.

Scorch fastness assessments revealed the most significant variations among the different dye structures and between the two application methodologies. The azo dye derivatives **2a** (X = H), **2c** (X = 3-Cl), and **2d** (X = 3-COOH) exhibited excellent scorch fastness ratings of 5 on both cotton and polyester substrates when applied via aqueous dyeing process, demonstrating exceptional thermal stability suitable for high-temperature textile processing operations. The use of a mixture of H_2_O-DMF in dyeing process resulted in marginally reduced scorch fastness for compounds **2b**,** 2c**, and **2e** (4–5 ratings), though compound **2c** maintained excellent performance (5) on polyester substrates. The dye contains 3-CHO group **2e** showed consistently good performance (4–5) across both substrates and application methods.

Light fastness evaluations revealed exceptional photostability for the entire dye series **2a-f**. All dyes achieve the maximum rating of 7–8 regardless of the dyeing in pure H_2_O and mixture of 5% DMF-95% H_2_O employed. This outstanding resistance to photochemical degradation represents a significant commercial advantage, particularly for textile applications involving prolonged sunlight exposure such as outdoor fabrics, automotive textiles, or window treatments.

The comparative assessment between dyeing in pure H_2_O and 5% DMF-95% H_2_O indicates that while both approaches yield commercially acceptable fastness properties, the aqueous system with dispersing agent generally provides marginally superior performance for most fastness categories. However, the dyeing in 5%DMF-95% H_2_O followed the same condition offers advantages in terms of dye dissolution and application simplicity, making the choice between two methods of dyeing dependent on specific processing requirements, available equipment, and the particular dye structure being employed. 

#### Color Strength and Dye Exhaustion Performance

The evaluation of color strength and dye exhaustion represents the commercial viability and economic efficiency of disperse dyes in textile applications. Color strength, expressed through the Kubelka-Munk K/S function, Eq. 1 [40], quantifies the light absorption capacity of dyed fabrics. Dye exhaustion percentage %E gives the efficiency of dye uptake from the dye bath, Eq. 2 [41], and it reflects the affinity between dye and fiber during the dyeing process. The comprehensive analysis of K/S values for the synthesized azo dyes **2a-f** provides valuable insights into structure-performance relationships and enables optimization of dyeing conditions for maximum color yield and process efficiency, Table 4.

**Table 4:** Reflectance, color strength and exhaustion of disperse dyes **2a-f**

Dyed Fabric		PE Fabric (H_2_O + dispersing agent)				PE Fabric (5%DMF- 95%H_2_O)			
		*R* (%)	K/S	K/S_sum_	%E	*R* (%)	K/S	K/S_sum_	%E
2-H	**2a**	14.81	2.45	55.97	76.09	14.45	2.53	38.65	65.35
2-F	**2b**	24.26	1.18	32.86	61.63	24.26	1.18	32.51	60.39
2-Cl	**2c**	15.04	2.40	61.12	78.76	16.14	2.18	61.06	78.60
2-COOH	**2d**	3.17	14.79	178.04	89.42	2.21	21.64	169.13	88.17
2-CHO	**2e**	12.64	3.02	46.74	67.67	14.79	2.45	36.28	63.80
2-CH_3_	**2f**	27.86	0.94	46.20	67.33	27.43	0.96	44.94	66.44

1 $$\:K/S\:=(1-{R)}^{2}/2R$$

Where *R* is the decimal fraction of the reflection of the dyed fabric; K the absorption coefficient; and S the scattering coefficient.

2 $$\:\%E=\frac{{C}_{1}-{C}_{2}}{{C}_{1}}*100$$

Where (C_1_) is the concentration of dye bath before and (C_2_) is concentration of dye bath after dyeing.

The reflectance measurements of polyester fabrics dyed with compounds **2a-f** revealed significant variations between the two dyeing methods and among different dye structures. The introduction of 3-COOH **2d** to the parent dye 2a (X = H) has the lowest reflectance (R) values with both dyeing methods (*R* = 3.17% in pure water and 2.21% in 5%DMF-95% H_2_O) indicating the darkest coloration and highest light absorption capacity among the series. This exceptional performance can be attributed to the presence of both phenolic hydroxyl and 3-COOH groups, which enhance dye-fiber interactions and potentially facilitate deeper dye penetration into the polyester structure. The 3-CH_3_ derivative **2f** registers points out the highest reflectance values (27.86% with pure H_2_O and 27.43% when dyeing is processing in 5% DMF-95% H_2_O suggesting lighter coloration despite maintaining reasonable dye uptake efficiency.

Color strength analysis, expressed by K/S values, Eq. 1, at the absorption maximum wavelength λ_max_, revealed substantial differences in dyeing performance across the dye of the nature of 3-substituent and between method conditions. The presence of 3-COOH **2d** showed remarkably high K/S values of 14.79 in pure H_2_O and an even more impressive 21.64 in 5% DMF-95% H_2_O, representing the highest color strength values in the entire series.

The summation of color strength values (K/S_sum), Eq. 3, across the visible spectrum provides a comprehensive measure of overall dyeing performance and color depth. The 3-COOH derivative **2d** has exceptional K/Ssum values of 178.04 in pure H_2_O dyeing and 169.13 when the dyeing process is run in 5% DMF-95% H_2_O, representing the highest cumulative color strength in the series. These values significantly exceed those of other compounds, confirming the superior dyeing performance of the 3-COOH derivative coupling component. Notably, dye **2d** exhibited a K/S of 21.64 in the 5% DMF-95% H₂O system, which corresponds to a ≈ 46.3% increase compared with its K/S of 14.79 in the pure water system, underscoring the significant positive effect of the DMF-containing bath on color uptake and depth. In contrast, the presence of 3-F substituent **2b** has lowest K/Ssum values with both dyeing methods, indicating poor dyeing performance regardless of the application technique.

3 $$\:K/{S}_{\mathrm{s}\mathrm{u}\mathrm{m}}\:=\:\:\sum\:_{390}^{700}{(\mathrm{K}/\mathrm{S})}_{\lambda\:}$$

#### Colorimetric Analysis of Dyes 2a-f: CIE Lab and LCh Color Space Evaluation

**Fig. 9:**
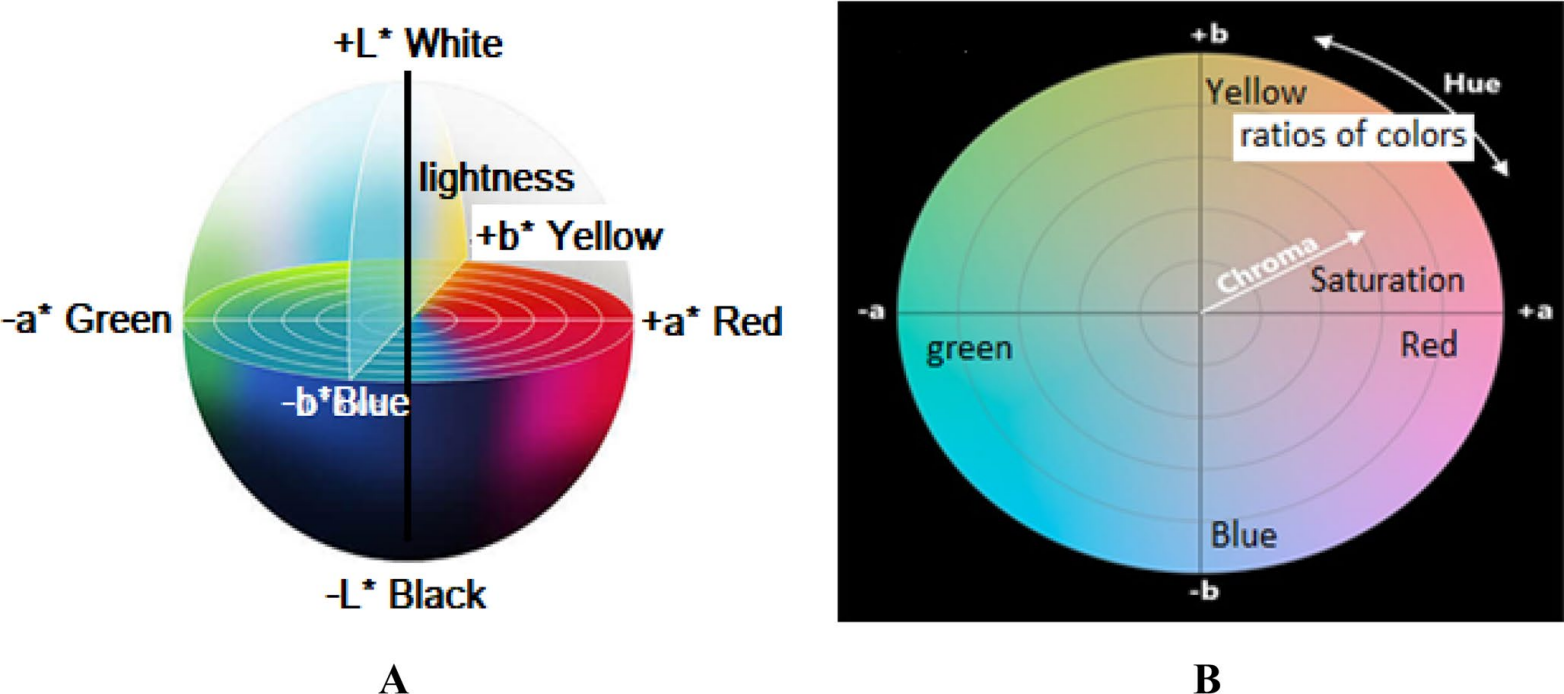
A) The CIE Lab color space, B) The LCh color space

The CIE Lab color space represents one of the most significant advances in colorimetry and color measurement science [42]. The system comprises three fundamental coordinates: L* (lightness), a* (red-green chromaticity), and b* (yellow-blue chromaticity). The L* parameter quantifies the perceived lightness of a color, ranging from 0 (absolute black) to 100 (perfect white). The a* coordinate defines the position along the red-green axis, where positive values indicate red tendencies and negative values represent green characteristics. Similarly, the b* coordinate describes the yellow-blue axis positioning, with positive values corresponding to yellow hues and negative values to blue tones. The colorimetric properties of dyed fabrics **2a-f** were obtained with a Hunter Lab DP-9000 Color-Spectrophotometer, Table 5. Brightness is the perception of the eyes on the brightness of the light source and the object surface. Brightness not only determines the degree of illumination of the object but also determines the reflection coefficient of the object surface, Fig. 9A. The LCh color space “L”, “C” and “h” are the three elements of color. ”L” stands for lightness, “C” is chroma, which is the degree of color saturation, and “h” is hue, which is the overall tendency of the color. These coordinates can be calculated, providing comprehensive color characterization essential for textile dyeing applications and quality control processes [43], Fig. 9B.

**Table 5:** Colorimetric data CIE Lab spaceof dyes **2a-f**

Dyed fibre	PE Fabric (H_2_O + dispersing agent)					Dyed fibre	PE Fabric (5%DMF-95% H_2_O)				
	L*	a*	b*	C*	h^o^		L*	a*	b*	C*	h^o^
2a	73.48	3.89	28.32	28.58	82.17	**2a**	78.61	1.35	24.08	24.12	86.79
2b	74.86	4.74	17.43	18.06	74.78	**2b**	75.13	4.28	17.55	18.07	76.31
2c	67.54	8.04	19.52	21.11	67.61	**2c**	43.06	8.09	14.71	16.79	61.18
2d	72.26	8.18	50.93	51.58	80.88	**2d**	72.06	4.33	47.55	47.75	84.79
2e	77.95	2.46	26.18	26.30	84.63	**2e**	77.84	1.57	20.31	20.37	85.59
2f	68.33	8.25	13.90	16.16	59.30	**2f**	67.92	8.67	11.93	14.75	53.98

The colorimetric evaluation reveals significant differences between the two dyeing methods of dyeing employed, Table 5. With respect to lightness L*, the dyeing method in pure water generally produces lighter colored fabrics, for dyes **2a** (X = H**)**,** 2b** (X = 3-F), **2d** (X = 3-COOH), and **2e** (X = 3-CHO), compared to the dyed fibre dyeing in 5% DMF-95% H_2_O, indicating enhanced brightness (lightness) and reduced color depth. However, dye **2c** (X = 3-Cl) presents a remarkable contrast, showing significantly darker coloration in 5% DMF-95% H_2_O (L* = 43.06) compared to the pure H_2_O (L* = 67.54), representing a substantial shift of approximately 24 units. This difference suggests that the 3-Cl derived dye **2c** exhibits enhanced substantivity and penetration in the organic solvent medium, resulting in deeper color development.

The a* values have consistent patterns across both dyeing methods, with all dyes maintaining positive values, indicating a universal tendency toward red-shifted hues. The b* coordinate analysis reveals that 5% DMF-95% H_2_O generally produces reduced yellow contributions compared to the pure H_2_O method, particularly evident in dyes **2a** (X = H), **2b** (X = 3-F), **2d** (X = 3-Cl), and **2e** (X = 3-CHO).

The measured chroma values (C*) reveal that the dyeing in pure H_2_O generally produces higher color saturation, particularly for dyes **2a** (X = H), **2d** (X = 3-COOH), and **2e** (X = 3-CHO). This enhanced saturation (intensity) in the pure H_2_O system may result from improved dye dispersion and uniform distribution throughout the fabric matrix. Conversely, dye **2c** (X = 3-Cl) shows significantly higher chroma in 5% DMF-95% H_2_O (C* = 16.79 vs. 21.11), correlating with its enhanced color depth in this medium.

Since the hue “h” value, express the overall tendency of the color, Table 5 indicates that the color of dyes **2a-f** range between red and yellow, while the h^o^ values indicate the ratio between these two colours. For example the ratio for dyed fibre **2a** in case of dyeing in pure H_2_O and 5% DMF-95% H_2_O is approximately the same and the remaining dyes **2b-f** follows the same behavior, while the h^o^ values for dyed fibre **2a-f** indicate that the ratio between the red and yellow colors in pure H_2_O and in % DMF-95% H_2_O are different depending on the 3-substituent.

### DFT Study

Density functional theory (DFT) calculations serve as a computational approach for elucidating the electronic properties and molecular behavior of organic dyes [44, 45]. The DFT studies the molecular descriptors namely HOMO-LUMO energy gaps ΔE, electronegativity (χ**)**, chemical hardness (η), softness (S) and electrophilicity (ω), dipole moment (D) indices. DFT studies can explain dye-fiber interactions and optimizing dye design for enhanced coloration performance. In this study, we employed DFT calculations at the B3LYP/6-31G(d, p) level to investigate the molecular descriptors of azo dyes **2a-f**, Table 6.

**Table 6:** Chemical descriptor parameters of dyes **2a-f**

Parameter	2a	2b	2c	2d	2e	2f
E_HOMO_ (eV)	−5.6487	−5.7317	−5.7815	−5.7809	−5.8084	−5.6027
E_LUMO_ (eV)	−2.1135	−2.2223	−2.2574	−2.2122	−2.2835	−2.0879
∆E (kcal/mol)	3.5352	3.5094	3.5241	3.5687	3.5249	3.5148
IP (eV)	5.6487	5.7317	5.7815	5.7809	5.8084	5.6027
EA (eV)	2.1135	2.2223	2.2574	2.2122	2.2835	2.0879
χ(eV)	3.8811	3.9770	4.0194	3.9966	4.0460	3.8453
µ (eV)	−3.8811	−3.9770	−4.0194	−3.9966	−4.0460	−3.8453
η(eV)	1.7676	1.7547	1.7620	1.7843	1.7624	1.7574
S(eV^− 1^)	0.5657	0.5699	0.5675	0.5604	0.5674	0.5690
ω(eV)	4.2607	4.5069	4.5844	4.4758	4.6441	4.2068
µ (D)	0.0099	2.0583	2.6626	3.1672	4.9366	0.5978

**Fig. 10:**
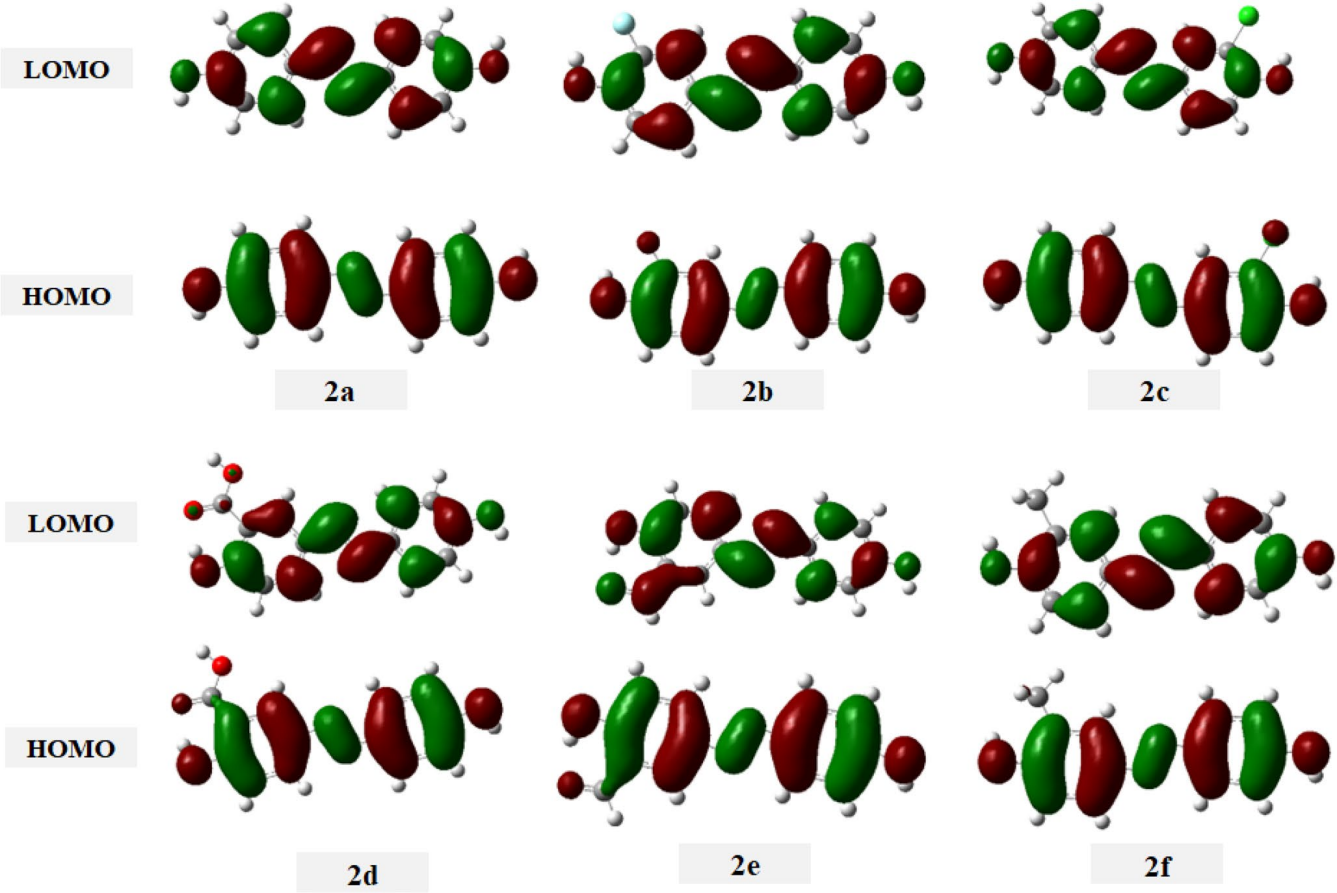
HOMO and LUMO of disperse azo dyes 2a-f

The DFT calculations performed on 4,4’-dihydroxy-3-substituted azobenzene dyes **2a-f** reveal significant variations in their electronic properties that directly correlate with their potential dyeing performance. Figure 10 represent HOMO and LUMO energy diagram which demonstrate that compounds **2a-f** exhibit similar patterns with electron clouds of the HOMO and LUMO are distributed throughout all the molecule due to the high conjugation system between two aryl rings through the azo group. The HOMO energies range from − 5.6027 eV (**2f**) to −5.8084 eV (**2e**, X = 3-CHO), indicating that dye **2f** (X = 3-CH_3_) possesses the highest electron-donating ability, while **2e** shows the greatest resistance to electron loss. Correspondingly, the LUMO energies vary from − 2.0879 eV (**2f**) to −2.2835 eV (**2e**), demonstrating the strongest electron-accepting ability.

The energy gap (ΔE) values, which are the best indicators of molecular reactivity and dye-fiber interaction strength, show relatively narrow variation ranging from 3.5094 kcal/mol (**2b**, X = 3-F) to 3.5687 kcal/mol (**2d**, X = 3-COOH), suggesting comparable reactivity profiles across the series, though **2b** exhibits slightly enhanced reactivity due to its smaller energy gap. 

Moreover, the substituent-dependent energy gap rationalize the observed spectral shifts. The HOMO-LUMO gap narrows (ΔE: 3.5094 → 3.5687 eV), which lowers the vertical excitation energy and produces a bathochromic shift. TD-DFT and experiment both confirm this trend (λ_max_ increases markedly for **2f)**, with the larger magnitude of the optical shift arising from excited-state relaxation, solvent stabilization and configuration mixing that are captured in the TD-DFT vertical excitations but not by the raw HOMO–LUMO difference alone. In contrast, electron-withdrawing substituents (such as formyl or carboxyl groups) withdraw electron density, stabilize the HOMO, and widen ΔE, causing hypsochromic shifts. 

The electronegativity (χ) values reveal that dye **2f** (X = 3-CH_3_) displays the lowest electronegativity (3.8453 eV), indicating potentially high dyeing behavior due to its enhanced electron-donating capacity, while **2e** (X = 3-CHO) shows the highest electronegativity (4.0460 eV). 

The global hardness (η) and softness (S) parameters demonstrate that **2d** (X = 3-COOH) possesses the highest hardness (1.7843 eV) and correspondingly the lowest softness (0.5604 eV^− 1^), suggesting reduced polarizability and potentially weaker dye-fiber interactions compared to other dyes in the series. Conversely, **2b** (X = 3-F) exhibits the highest softness (0.5699 eV^− 1^), indicating enhanced molecular flexibility and stronger potential for dye-substrate interactions.

The electrophilicity index (ω) values show that **2e** (X = 3-CHO**)** has the highest electrophilicity (4.6441 eV), suggesting high electron-accepting capability and potential for effective dyeing, while **2f** (X = 3-CH_3_) displays the lowest value (4.2068 eV).

Particularly noteworthy are the dipole moment variations, where **2e** (X = 3-CHO) exhibits the highest dipole moment (4.9366 D), this high electrophilicity and elevated dipole moment of **2e** provide theoretical support for enhanced dye-fiber interactions for example, by increasing polarity-driven hydrogen bonding and electrostatic attraction to polyester ester groups and therefore help explain the compound’s improved practical fixation. While **2a** (X = H) shows minimal polarity (0.0099 D). The structural modifications introduced by different phenolic couplers significantly influence the electronic properties, with the aldehyde group in **2e** (X = 3-CHO) creating the most polarized system and enhanced electrophilicity, the fluorine substitution in **2b** (X = 3-F) providing optimal softness characteristics, and the methyl substitution in **2f** (X = 3-CH_3_) generating favorable electron-donating properties, collectively suggesting that the dyeing performance hierarchy would likely follow the order **of 2e** (X = 3-CHO) **> 2b** (X = 3-F) **> 2c** (X = 3-Cl**) ≈ 2 d (**X = 3-COOH**) > 2f (**X = 3-CH_3_**) > 2a** (X = H) based on the combination of electronic parameters favoring strong dye-fiber interactions and effective charge transfer processes.

The molecular electrostatic potential surfaces (MEP) of dyes **2a-f** provide their charge distribution patterns and potential reactive sites, which are fundamental for explaining their interaction mechanisms with textile substrates.

**Fig. 11:**
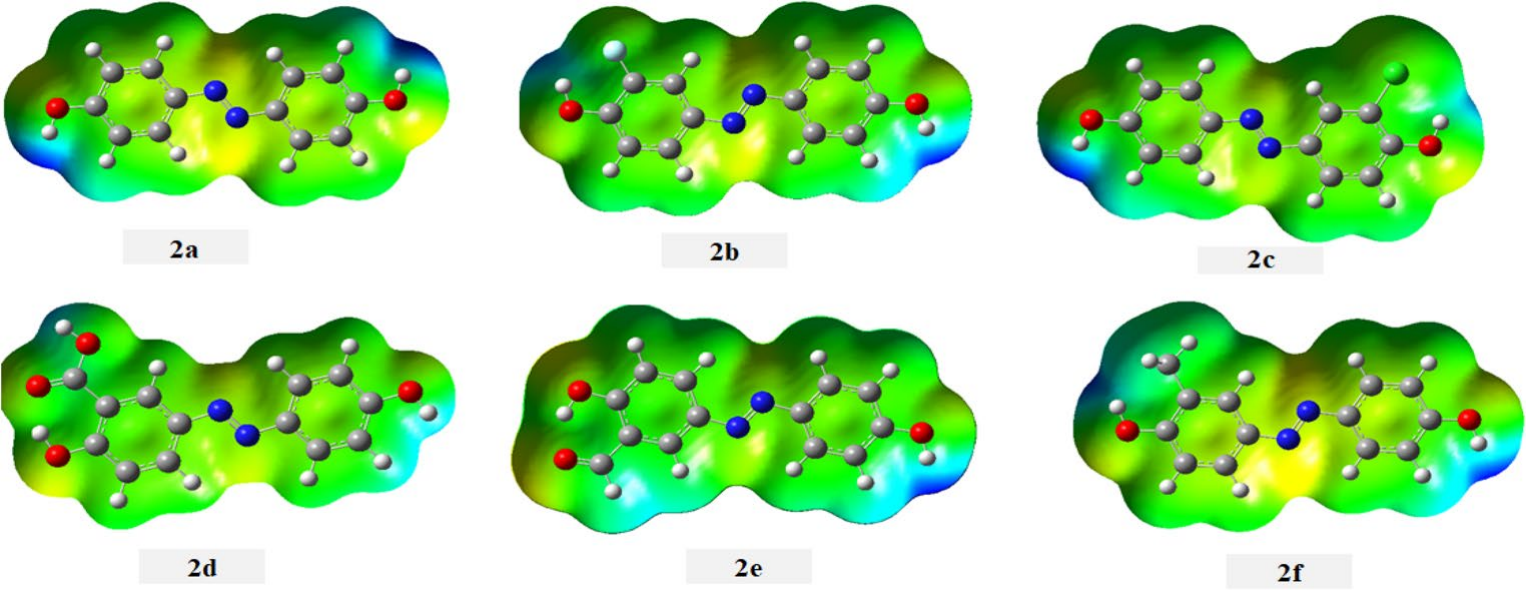
The MEP mapping of dyes 2a-f

The MEP mapping reveals distinct electrostatic characteristics across different molecular regions that directly influence the dyeing behavior and substrate affinity of azo dyes **2a-f**, Fig. 11. The blue colour region observed on the hydroxyl hydrogen, representing electron-deficient areas that are susceptible to nucleophilic attack and can participate in hydrogen bonding interactions with fiber molecules. i.e. it facilitates the formation of intermolecular hydrogen bonds with polar functional groups present in textile fibers. Thereby, this type of bonding enhance dye-substrate adhesion and color fastness. The yellow regions localized over the azo linkage (-N = N-) can possess intermediate electrostatic potential, due to the delocalized π-electron through the chromophoric –N = N- unit and in turn is responsible for the dyes’ color properties. The green areas distributed throughout the phenyl rings indicate neutral to slightly negative electrostatic potential, corresponding to regions of moderate electron density that contribute to the overall stability of the molecular framework. These aromatic regions, with their π-electron clouds, can engage in π-π stacking interactions with aromatic components of textile fibers, providing additional anchoring points that strengthen the dye-fiber association.

## Conclusion

This study successfully synthesized and characterized six 4,4’-dihydroxy-3-substituted azo disperse dyes **2a-f** derived from 4-aminophenol. These dyes have demonstrating excellent correlation between theoretical predictions and experimental observations. DFT calculations at the B3LYP/6-31G(d, p) level revealed significant variations in electronic properties, DFT analysis identified **2e** as having the highest electrophilicity (4.6441 eV) and dipole moment (4.9366 D), providing theoretical support for enhanced dye-fiber interaction; experimentally, **2d** delivered the highest color strength (K/S = 21.64 in 5% DMF–95% H₂O), representing a ~ 46.3% increase over the pure water system (K/S = 14.79). TD-DFT calculations showed remarkable agreement with experimental UV-visible absorption data, with deviations typically less than 10 nm, validating the computational approach for predicting optical properties. The synthesized dyes have pH-responsive behavior with substantial bathochromic shifts (75–80 nm) under basic conditions, suggesting potential applications as colorimetric indicators. The colorimetric evaluation indicates that the dyeing method in pure water generally produces lighter colored fabrics, compared to the dyed fibre resulted from dyeing in 5% DMF-95% H_2_O. Also, the dyed fibre color ranged between red and yellow color depending on the substituent and independent on the medium condition of dyeing process. Dyeing performance evaluation revealed that the dye **2d** (X = 3-COOH) has exceptional color strength values (K/S = 21.64 in 5% DMF-95% H_2_O), while all dyes have excellent light fastness ratings (7–8). Comparative analysis between dyeing in pure H_2_O-dispersing agent and 5% DMF-95% H_2_O processes showed that both methods of dyeing yield commercially acceptable results, taking into consideration the dyeing in pure H_2_O system providing marginally superior fastness properties while 5% DMF-95% H_2_O offers advantages in dye dissolution and application simplicity.

## Supplementary Information

Below is the link to the electronic supplementary material.

Supplementary Material 1 (DOCX 524 KB)

## Data Availability

The data used and analyzed during the current study are available inside the manuscript or in the supplementary material.
