# Supplementary material for: Synthesis, DFT Analysis, and Dyeing Performance of 4,4’-Dihyroxy-3-Substituted Azobenzene Disperse Dyes with Comparative Evaluation of Aqueous and DMF-Based Application Systems
Source: J Fluoresc. 2026 Apr 20;36(5):3283–98. doi: 10.1007/s10895-026-04754-z (PMC13226321; doi:10.1007/s10895-026-04754-z)
Supplement: Supplementary file 1 — Supplementary Material 1 (DOCX 524 KB) [file 10895_2026_4754_MOESM1_ESM.docx]

**Synthesis, DFT Analysis, and Dyeing Performance of 4,4’-Dihyroxy-3-Substituted Azobenzene Disperse Dyes with Comparative Evaluation of Aqueous and DMF-Based Application Systems**

Mohamed A. El-Rahman^1^, Mai S. Alsubaie^1^, Mohamed A. El-Atawy^1,2^, Hussam Y. Alharbi^2^, Majed S. Aljohani^2^, Saad Alrashdi^3^, Alaa Z. Omar^1,*^, Ezzat A. Hamed^1^ and Reham O. El-Zawawy^4^

1. Chemistry Department, Faculty of Science, Alexandria University, Alexandria 21231, Egypt.
2. Chemistry Department, College of Sciences, Taibah University, Yanbu 46423, Saudi Arabia
3. Department of Chemistry, College of Science, Jouf University, Sakaka 72341, Aljouf, Saudi Arabia
4. Chemistry Department, Faculty of Science, Damanhour University, Damanhour 22511, Egypt

* Correspondence: alaazaki@alexu.edu.eg

**Instruments and Apparatus:**

Melting points were determined by MEL-TEMP II melting point apparatus in open glass capillaries. The IR spectra were recorded as potassium bromide (KBr) discs on a Perkin-Elemer FT-IR (Fourier-Transform Infrared Spectroscopy), Faculty of Science, Alexandria University. The NMR spectra were carried out at ambient temperature (~25 ºC) on a (JEOL) 500 MHz spectrophotometer using tetramethylsilane (TMS) as an internal standard, NMR Unit, Faculty of Science, Alexandria University. Elemental analyses were analyzed at the Regional Center for Mycology and Biotechnology, Al-Azhar University, Cairo, Egypt.

**The experiment of color fastness properties test**

ASTM (American Society for Testing and Materials) and AATCC (American Association of Textile Chemists and Colorists) developed some standards for testing the color fastness properties that were applied in this research for testing the samples.

**Fastness to washing.**

The wash fastness properties of the fabric samples were tested using the ASTM D435-42 Standard with the Launder Meter TF418. A sample of dyed PET material was sewn between two white cotton fabrics, with equal lengths of 5 x 2.5, and the color was assessed using the worldwide grey scale (1 represents poor and 5 represents excellent).

**Fastness to light.**

The light-fastness properties of the fabric samples were tested using the AATCC TM16.1 Standard with the Light Fastness Tester TF421. The fabric was exposed to high-energy radiation “Q-SUN Xenon Test Chamber, QLAB, USA” in a “fade-o-meter” for 18-20 hours (scale: 1 for poor and 8 for outstanding).

**Fastness to perspiration**

The perspiration fastness properties of the fabric samples were tested using the AATCC TM15 Standard with the Perspirometer TF416A.

**Color measurements**

The colorimetric properties of dyed PET fabrics were obtained using a Hunter Lab Ultra Scan PRO (Reston, Virginia, USA) in terms of CIELab values (L*, a*, b*, c*, h°) using a standard illuminant D65 and 10° observer with specular radiation excluded on a Minolta CM-3600d visible spectrophotometer

According to this system, three basic tristimulus components of color, namely, hue (h°), chroma (C*) (also referred to as saturation), and Lightness (L*) (also referred to as luminance), were measured. The hue angle was measured from 0 to 360°. The values of the two coordinates, a* and b*, were also determined. L* represents the lightness or darkness of a color (L*= 100 for white and L*= 0 for black), whereas a* = red to green (+a* = redder, -a* = greener), and b* = yellow to blue (+b* = yellower, −b* = bluer), and where the two 'color' axes intersect = neutral gray.

The chroma (c*) and hue angle (h°) were measured using equations 1 and 2, respectively.

$Chroma C^{*}= \sqrt{a^{*2}+b^{*2}}$ Eq. 1

$Hue angle h^{o}={tan}^{-1}\left( \frac{b^{*}}{a^{*}} \right)$ Eq. 2

ΔH* stated color hue difference values. ΔL* stated the differences in the lightness values. The values of ΔL*, ΔC* and ΔH* are calculated by the following equations 3-5:

ΔL* = L*(standard value, X = H) – L*(substituted value) Eq. 3

ΔC* = c*(standard value, X = H) – c*(substituted value) Eq. 4

ΔH* = h*(standard value, X = H) – h*(substituted value) Eq. 5

**Computational methodology**

All geometry optimizations were performed using the density functional theory (DFT) at the B3LYP functional. The calculations were carried out using the GAUSSIAN 09 package. A full optimization was performed up to a higher basis set denoted by 6-31G(d,p) because this basis set and The maximal wavelengths (ʎmax) were systematically investigated using TD-DFT/B3LYP/6-31G(d,p) method, based on the optimized ground state.


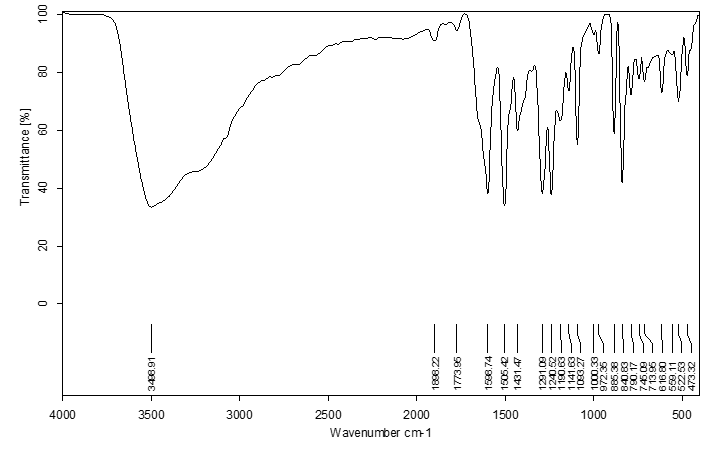


IR (KBr) spectrum of (*E*)-2-fluoro-4-((4-hydroxyphenyl)diazenyl)phenol **2b**

^^

^1^H NMR (DMSO-*d6*) spectrum of (*E*)-2-fluoro-4-((4-hydroxyphenyl)diazenyl)phenol **2b**

^
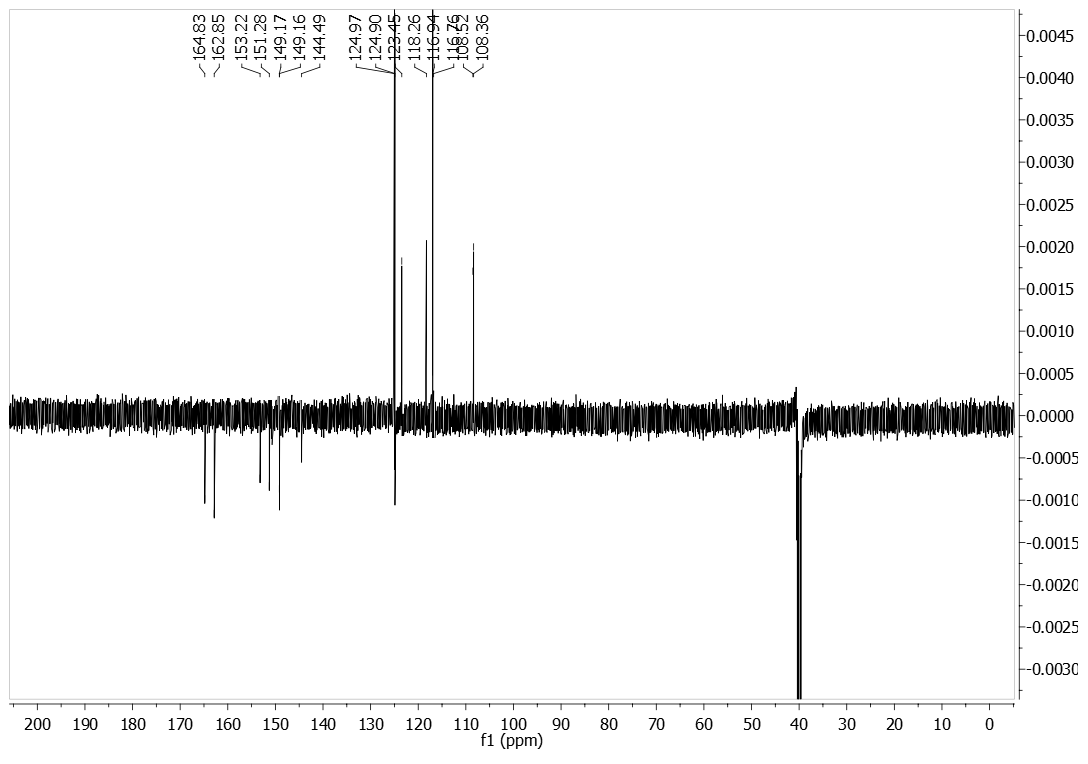
^

^13^C NMR (DMSO-*d6*) of (*E*)-2-fluoro-4-((4-hydroxyphenyl)diazenyl)phenol **2b**

^13^C NMR (DMSO-*d6*) of (*E*)-2-fluoro-4-((4-hydroxyphenyl)diazenyl)phenol **2b**

**
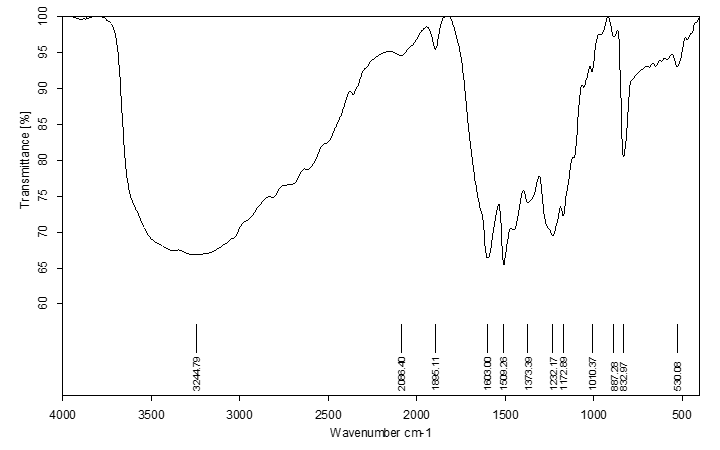
**

IR (KBr) spectrum of (*E*)-2-chloro-4-((4-hydroxyphenyl)diazenyl)phenol **2c**

**_
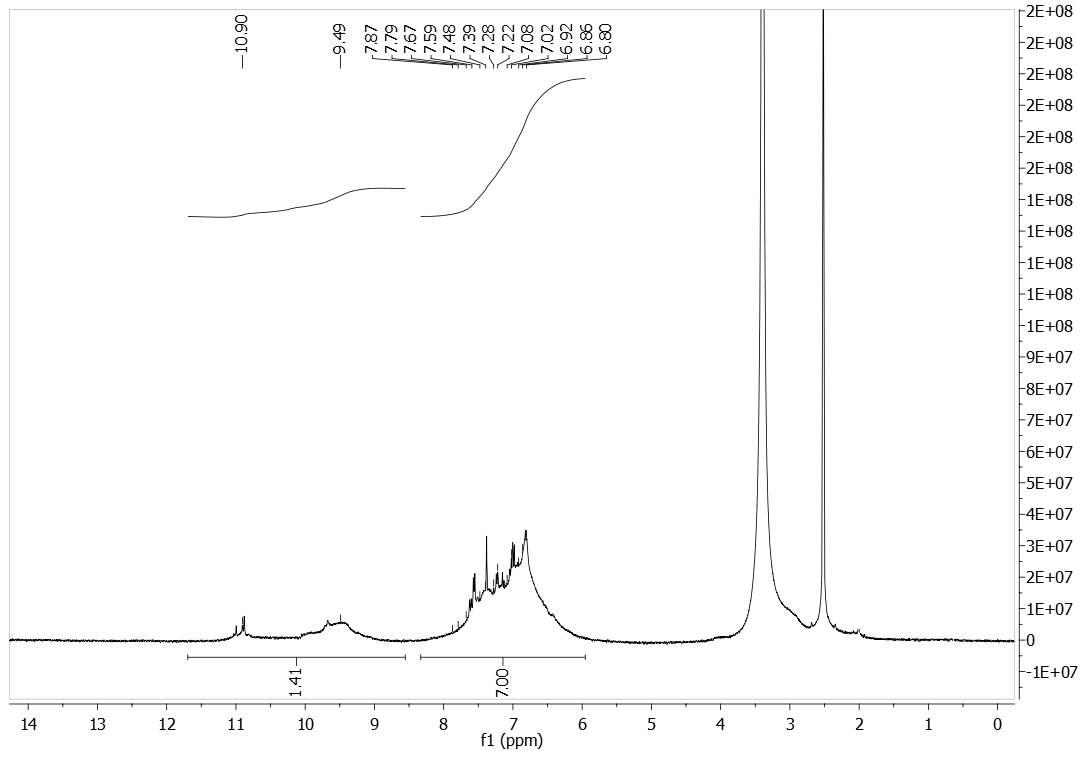
_**

^1^H NMR (DMSO-*d6*) spectrum of (*E*)-2-chloro-4-((4-hydroxyphenyl)diazenyl)phenol **2c**

**
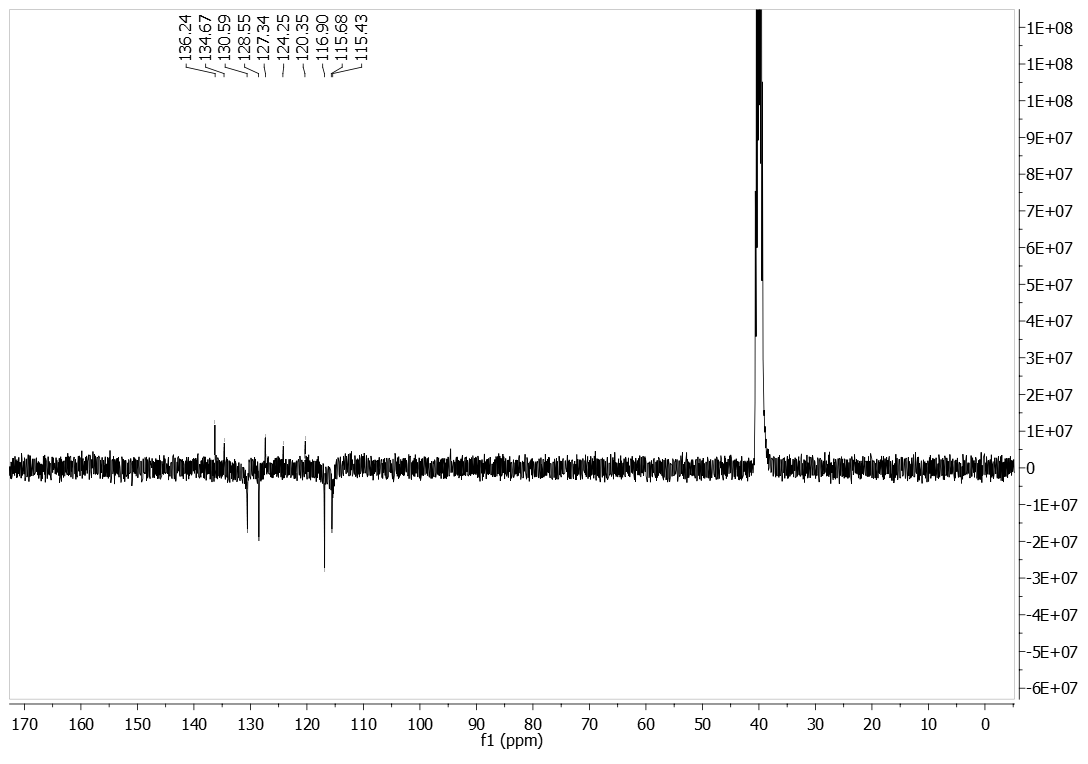
**

^13^C NMR (DMSO-*d6*) of (*E*)-2-chloro-4-((4-hydroxyphenyl)diazenyl)phenol **2c**


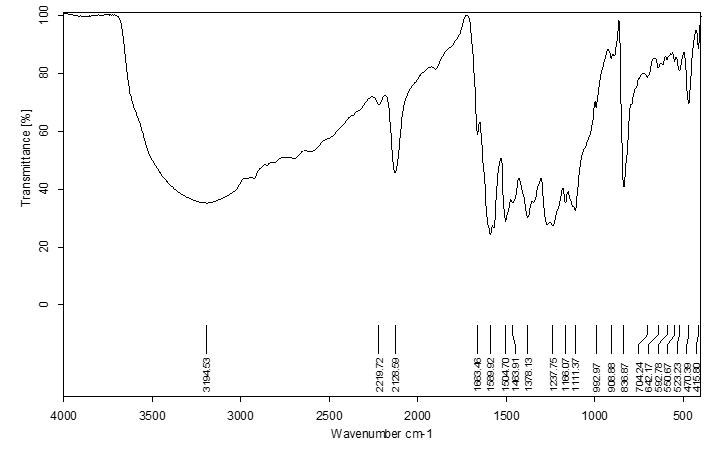


IR (KBr) spectrum of (*E*)-2-hydroxy-5-((4-hydroxyphenyl)diazenyl)benzoic acid **2d**

**
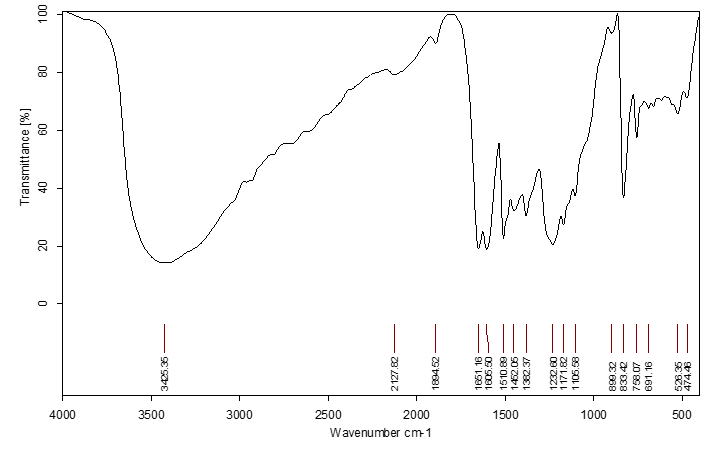
**

IR (KBr) spectrum of (*E*)-2-hydroxy-5-((4-hydroxyphenyl)diazenyl)benzaldehyde **2e**

**_
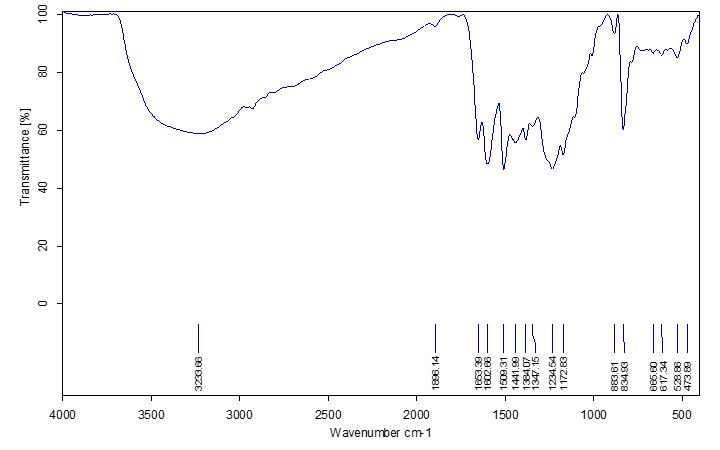
_**

IR (KBr) spectrum of (*E*)-4-((4-hydroxyphenyl)diazenyl)-2-methylphenol **2f**


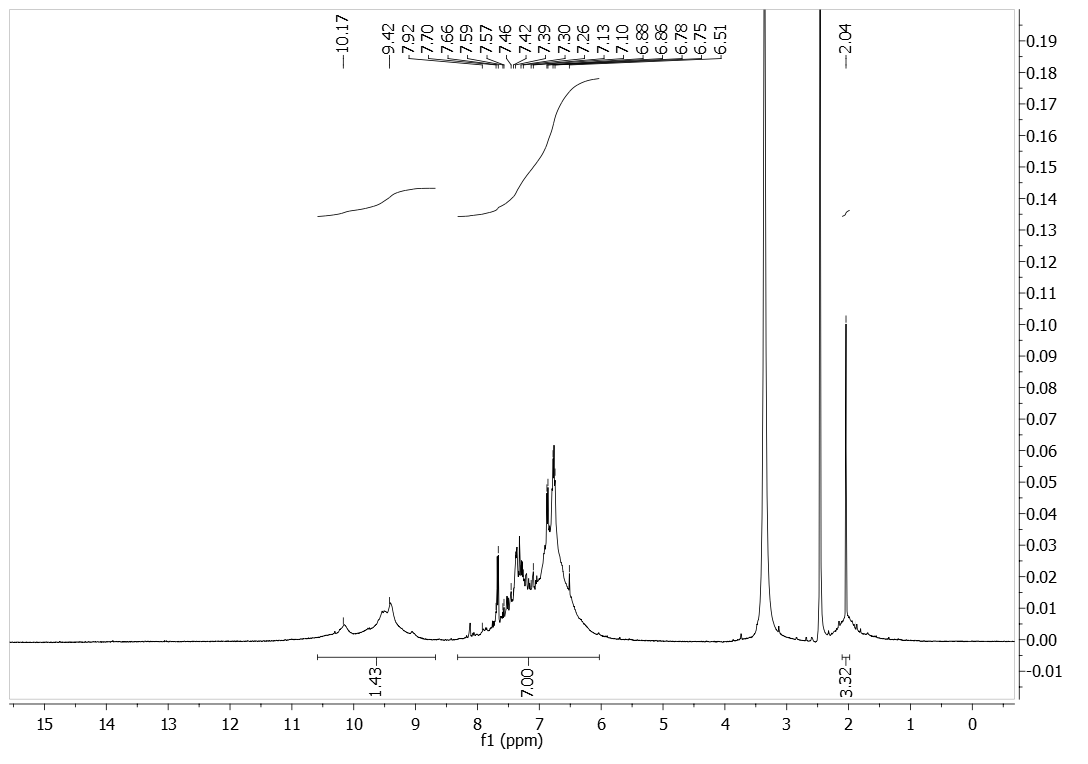


^1^H NMR (DMSO-*d6*) spectrum of (*E*)-4-((4-hydroxyphenyl)diazenyl)-2-methylphenol **2f**


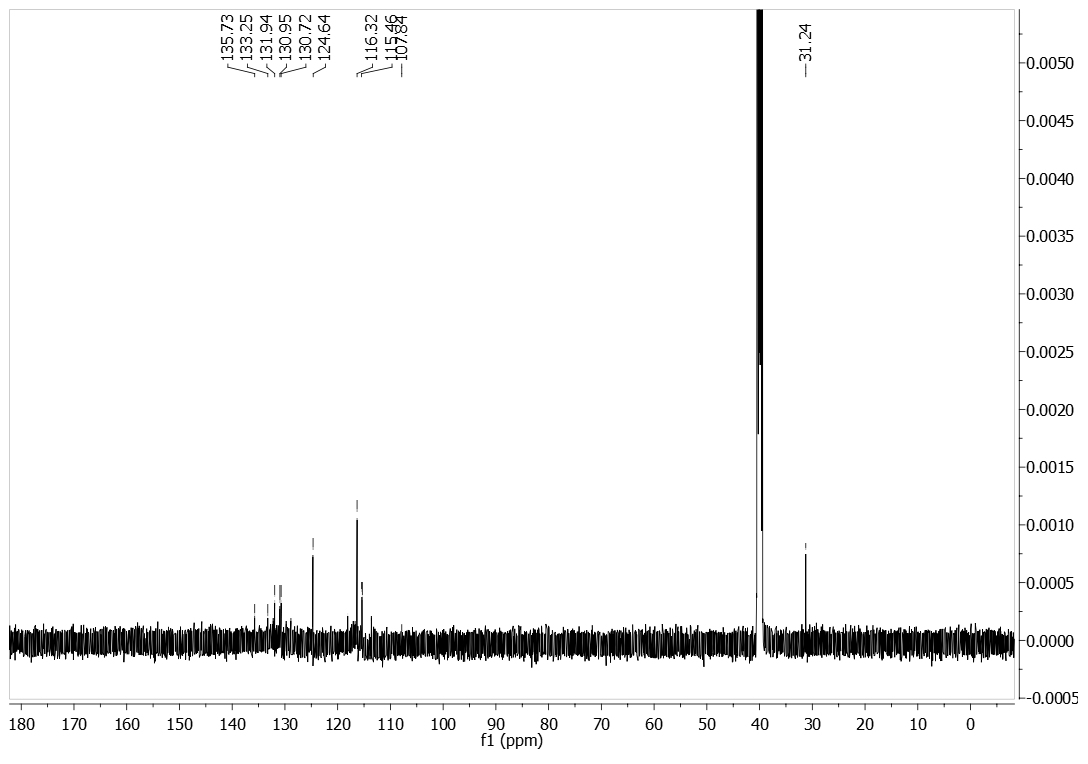


^13^C NMR (DMSO-*d6*) of (*E*)-4-((4-hydroxyphenyl)diazenyl)-2-methylphenol **2f**
